# Fluid Mechanics of Mosaic Ciliated Tissues

**DOI:** 10.1103/PhysRevLett.127.198102

**Published:** 2021-11-05

**Authors:** Francesco Boselli, Jerome Jullien, Eric Lauga, Raymond E. Goldstein

**Affiliations:** 1 Department of Applied Mathematics and Theoretical Physics, Centre for Mathematical Sciences, University of Cambridge, Cambridge CB3 0WA, United Kingdom; 2 Wellcome Trust/Cancer Research UK Gurdon Institute, Tennis Court Road, Cambridge CB2 1QN, United Kingdom; 3 Department of Zoology, University of Cambridge, Cambridge CB2 1QN, United Kingdom; 4 Inserm, Nantes Université, CHU Nantes, CRTI-UMR 1064, F-44000 Nantes, France

## Abstract

In tissues as diverse as amphibian skin and the human airway, the cilia that propel fluid are grouped in sparsely distributed multiciliated cells (MCCs). We investigate fluid transport in this “mosaic” architecture, with emphasis on the trade-offs that may have been responsible for its evolutionary selection. Live imaging of MCCs in embryos of the frog *Xenopus laevis* shows that cilia bundles behave as active vortices that produce a flow field accurately represented by a local force applied to the fluid. A coarse-grained model that self-consistently couples bundles to the ambient flow reveals that hydrodynamic interactions between MCCs limit their rate of work so that they best shear the tissue at a finite but low area coverage, a result that mirrors findings for other sparse distributions such as cell receptors and leaf stomata.

An indication of the importance of fluid mechanics in biology is the remarkable degree to which the structure of eukaryotic cilia has been conserved over the past billion years [1,2]. These hairlike appendages provide motility to microorganisms [3,4] but also direct fluid flow inside animals during development [5–7] and in mature physiology in areas from the reproductive system [8] to the brain [9]. The two extremes of this organisimal spectrum have a fundamental distinction. In unicellulars like *Paramecium*, cilia are uniformly and closely spaced on the cell surface [10], while in animals they are often grouped together in dense bundles on multiciliated cells (MCCs) [11] that are sparsely distributed on large epithelia, as in the trachea and kidney [12,13]. This difference reflects the need in animal tissues to share surface area with cell types having other roles, such as mucus secretion.

The workings of cilia bundles and the significance of their sparse “mosaic” pattern for fluid transport have only begun to be investigated, primarily limited to *in vitro* or *ex vivo* studies [14–16]. Here we address the fluid mechanics of mosaic tissues using embryos of the amphibian *Xenopus laevis*. The cilia covering the epidermis drive a flow that sweeps away pathogens, preventing infection, and possibly contributing to oxygenation (Fig. 1) [17]. To date, the flow has served as a readout of cilia beating in the study of tissue patterning and cilia disorders [18–20]; here we take advantage of the geometry of *Xenopus* embryos to obtain side views of cilia bundles and quantify the flows they drive. As those cilia collectively sweep through cycles consisting of an extended “power” stroke and compact “recovery” stroke close to the surface [21], the flow within each bundle appears as an active vortex. While the flow driven by a single such vortex decays quickly with distance from the skin, we show that long range contributions of other bundles slows the decay of this *endogenous* flow and determines the shear stress at nonciliated cells. From measurements of beating changes induced by *exogeneous* flows, we determine the linear self-consistent relation describing the dissipative coupling between forces applied by bundles and the flows they generate; we find that bundles, as a collective, most efficiently shear the intervening tissue at the observed low area coverage. These results thereby suggest an explanation for the low area coverages observed in nature.

The *Xenopus* epidermis has strong similarities with human mucociliary epithelia [17]. The sparsely located MCCs whence emanate hundreds of cilia that drive a homogeneous anterior-to-posterior (A-P) flow (Fig. 1) are surrounded by nonciliated cells: “goblet cells” that cover most of the tissue secreting mucus-like material [24], mosaically scattered small cells [25,26] secreting serotonin vesicles that modulate the ciliary beat frequency [27], and ionocytes transporting ions important for homeostasis. Having evolved much earlier, *Xenopus* epidermis is simpler and more accessible than most vertebrate mucociliary epithelia. Unlike in airways, the fluid domain extends far above the cilia, and thus it is a model for fundamentals of mucociliary systems and their evolution.

Wild-type *Xenopus laevis* embryos were obtained via *in vitro* fertilization [28,29], and grown in 0.1× modified Barth’s saline at room temperature (or 15 °C to reduce the growth rate, if required). They were imaged at stage 28 [23] after treatment with 0.01% Tricaine to avoid twitching without affecting cilia dynamics [20]. Stage 28 embryos lie on one of their flat flanks, providing a side view of cilia in ventral MCCs (Fig. 1), whose power strokes are in the A-P direction (left to right in figures) so cilia and the flows stay mostly within the focal plane.

In flow chamber experiments, embryos were perfused with a peristaltic pump while in a Warner Instruments chamber (RC-31A): a 4 mm × 37 mm channel cut into a 350 *μ*m thick silicon gasket sandwiched between two coverslips. The anterior region of interest was > 2 mm away from the chamber wall, and the A-P axis parallel to the main direction of the perfusing flow. Brightfield images of cilia and 0.2–0.5 *μ*m tracers (mass fraction ~0.01%) were acquired at 2000 frames/ s for ≥ 1 s by a high speed camera (Photron Fastcam SA3) on an inverted microscope (Zeiss Axio Observer) with a long distance 63× objective (Zeiss LD C-Apochromat). Images were filtered by subtracting their moving average. Flow fields **u** = (*u, v, w*) were estimated by particle image velocimetry (PIVlab) and averaged over time. Pulsatility of the flow is low (fluctuations < 10% of the mean [29]), so we treat the flow as steady.

We first summarize important length scales and time-scales. MCCs are spaced 40–80 *μ*m apart and uniformly distributed with density 𝒫 ≈ 2.7 ± 0.73 × 10^−4^ *μ*m^−2^, from which we define a typical spacing $d=\sqrt{1/𝒫}\;\sim \;61$ *μ*m. With 𝓁 = 14.52 ± 0.21 *μ*m the cilia length, the average cellular area of 287 ± 11 *μ*m^2^ is ~𝓁^2^, and the coverage fraction *ϕ* ≈ 0.075 ± 0.02 is ~ (𝓁/*d*)^2^. Cilia beat at *f* ~ 20–30 Hz; during a power stroke their tips move ~2𝓁 in half a period, reaching speeds *V* ~ 4*f𝓁* ~ 1 mm/ s. The Reynolds number *V𝓁* */ ν* ~ 0.01 (with *ν* the kinematic viscosity of water) is in the Stokes regime.

Despite the tendency of nearby cilia to synchronize [4], those in an MCC are not in phase; a snapshot [Figs. 1(a) and 2(a)] shows cilia throughout the beat cycle [29], generating vorticity **ω**||**e**_*y*_ perpendicular to the beating plane. Each MCC is thus an *active vortex* [Figs. 2(b),2(c)]: *ω* can exceed ~150 s^−1^ ~ 2*V* / *𝓁*, is colocalized with the cilia, and rapidly diffuses at larger *z* as the flow becomes parallel to the skin. Above nonciliated cells, there is a shear flow for *z <* 𝓁, while for *z > d* the discreteness of the MCCs is washed out and *u* is independent of *x* (Fig. S3 [29]) and falls off slowly with *z* [Figs. 2(e),2(f)].

A first step toward understanding the coupling between cilia and flow involves quantifying the contribution of a single MCC. We introduce a boundary ∂Ω_*c*_ enclosing the volume Ω_*c*_ of the active vortex [Fig. 2(a)], and extrude it in *y* ∈ [−10 *μ*m; 10 *μ*m], the measured size of the vortex. The far-field flow from an isolated cilium is well approximated by that of a point force [34], and the velocity **u**_*s*_ driven by a point force **f** at *s* next to a no-slip wall is **u**_*s*_(**x**) = **f** · **S**(**x**, **s**) [29], with **S** the Blake tensor [35]. We model the bundle with *N* point forces **f**_*n*_ = (*f*_*n,x*_, 0, *f*_*n,z*_) at **s**_*n*_ = (*x*_*n*_, *y*_*n*_, *h*_*n*_) in Ω_*c*_, from which we define a single effective lateral force *F* = *𝓁*^−1^ *Σ*_*n*_ *h*_*n*_*f*_*n*,*x*_, applied at *z* = 𝓁 that drives a lateral flow *u*_*s*_ matching the far field of the entire bundle. This also equals the far-field velocity for a local moment (rotlet) Γ = 2𝓁*F* [36].

We first use an envelope approach [37,38] in which the cilia tips determine the flow, and find **f**_*n*_ by fitting the measured velocity on ∂Ω_*c*_ and a no-slip boundary at *z* = 0 [Fig. 2(d)]. The flow driven by these forces, **u**_0_ (**x**) ≈ Σ_*n*_ **f**_*n*_ · **S**( **x**, **s**_*n*_), is in an otherwise quiescent fluid. This fit yields a force *F*_0_ ≃ 209 ± 14 pN. For *z >* 2𝓁 and *x =* 0, the component *u*_0_ falls off like the flow *u*_*s*_ (*z*) ~ 3*F*_0_𝓁^2^ /4*πμz*^3^ above a single point force [35] (Fig. S2 [29]). The data [Fig. 2(e)] show a much weaker decay, which we now show arises from contributions of distant MCCs.

The simplest model for the *ambient flow u*_*a*_ for *z > d* assumes a uniform distribution of *x*-directed forces *F*, one for each MCC, with area density *𝒫*. In cylindrical coordinates (*ρ, θ, z*) centered on a bundle, the lateral flow due to any one force is $u_{s}(x)\approx F{\tilde{S}}_{xx}$, where ${\tilde{S}}_{xx}$ is the far-field limit of *S*_*xx*_,

(1) $${\tilde{S}}_{xx}(\rho ,\theta ,z)=\frac{3\ell }{2\pi \mu }\frac{z\rho^{2}\cos^{2}\theta }{{\left(\rho^{2}+z^{2}\right)}^{5/2}}.$$

Integrating up to a radius Λ that represents finite embryo size and subtracting that from the bundle at the origin, taken as a distribution of radius *d*/2, we obtain

(2) $$\begin{array}{l} u_{a}(z)=PF\int_{0}^{2\pi }\int_{d/2}^{\Lambda }\rho d\rho d\theta {\tilde{S}}_{xx}(\rho ,\theta ,z)\\ \;=U[G(z/\Lambda )-G(2z/d)], \end{array}$$

where *G*(*χ*) = 1 – (3*χ + * 2*χ*^3^)/2(1 + *χ*^2^)^3/2^ and *U* = *𝒫Fl / μ*. *G* decreases monotonically from *G*(0) = 1 to *G*(∞) = 0. For any fixed *z* ≫ *d*, as the organism size Λ → ∞, *χ* → 0, giving a *z* independent flow with speed *U* [39], while for any fixed Λ, *u*_*a*_ vanishes as *z*/Λ → ∞.

A fit of *u*_*a*_ to the data in Fig. 2 yields *U=* 0.63 ± 0.05 mm/ s and Λ = 308 ± 30 *μ*m [29], and using the observed *𝒫* ≈ 2.6 ± 0.5 × 10^−4^ *μ*m^−2^ we obtain the farfield estimate *F* ≃ 163 ± 35 pN. The fact that *u*_*a*_ ~ *u* for *z > d* confirms the slow decay above a bundle is established by distant MCCs [Figs. 2(e),2(f) and Fig. S3 in the Supplemental Material [29]). Direct summation of a lattice of point forces yields nearly identical results, validating the continuum approximation (Fig. S4 [29]).

Thus, we may model the flow as **u**(**x**) ≈ **u**_*c*_ (**x**) + *u*_*a*_**e**_*x*_, where **u**_*c*_ (**x**) ≈ Σ_*n*_ **f**_*n*_ · **S**(**x**, **s**_*n*_) is the local contribution, with the **f**_*n*_ found by fitting *u* − *u*_*a*_. We obtain *F* ≃ 162 ±30 pN, confirming the far-field estimate. Figures 2(e),2(f) show that the *u*_*c*_ + *u*_*a*_ gives an excellent fit to the data for *z >* 𝓁, above and between bundles.

The magnitude of *F* can be compared to the average lateral force generated by the power stroke of a cilium estimated from resistive force theory [40] as *f* ~ *ζ*_⊥_ 𝓁*V* / 12 ≃ 3 pN [29], where $\zeta_{⊥}=4\pi \mu /|\ln(\sqrt{e}\varepsilon )|$ is the transverse drag coefficient and *ε* ~ 75 is the cilium aspect ratio. Thus, a bundle contributes with ~50–60 cilia, about half the typically ~100 cilia in an MCC, reflecting force cancellations from phase shifts between cilia.

To probe the ciliary response to flow, we exposed the epidermis to an exogenous shear flow ${\dot{\gamma }}_{e}ze_{x}$ (Fig. 3). When ${\dot{\gamma }}_{e}=0$, the cilia drag the fluid, generating a negative shear $\dot{\theta }(0)=\partial u(z)/{\partial z|}_{\ell }\sim -23\;{\text{s}}^{-1}$ at the tips. Pumping fluid in the same direction, $\dot{\theta }$ decreases linearly with ${\dot{\gamma }}_{e}$, while the velocity *V* at the tips increases, but much more slowly. The rate of work above the cilia tips $W\propto -\dot{\theta }V$ thus decreases almost at the same rate as $\dot{\theta }$, and for ${\dot{\gamma }}_{e}\ell /V(0)>0.3$ becomes negative, consistent with a dissipative bundle. This reflects, in part, the dense packing of cilia in MCCs; the weak coupling is consistent with weak entrainment of dense bundles of ependymal cilia by exogenous flow [41].

Figure 3(c) shows that the lateral velocity $u\left(z;{\dot{\gamma }}_{e}\right)$ above the bundle (*z* < 2.5*𝓁*) is well fit by$u\left(\;z;{\dot{\gamma }}_{e}\right)\approx Cu(z;0)+{\dot{\gamma }}_{e}z$. Here, *C* ≈ *F/F*( 0), as confirmed by the above calculations, where *u*_*c*_ ≈ (*F / F*_0_) *u*_0_ for *z >* 𝓁 [Fig. S3(b) in Ref. [29] ]. It follows that *V* and force are coupled as $V=[F/F(0)]V(0)+{\dot{\gamma }}_{e}\ell$, and the slopes of the curves *F / F* (0) and *V / V* (0) versus ${\dot{\gamma }}_{e}\ell /V(0)$ are −*α*, and 1 − *α*, with *α* an empirical parameter. If *α =* 1, the bundle compensates for ${\dot{\gamma }}_{e}$ and the velocity at the tips is preserved, whereas if the force were constant (*α =* 0), the bundle supplies no resistance, and the ambient flow simply adds to that of the bundle. The measured slope *α* ≈ 0.76 ± 0.06 (Fig. 3) confirms resistive behavior.

The response *F* − *F*(0) of the bundle to ${\dot{\gamma }}_{e}$ can be parametrized by a drag coefficient (viscosity × length), analogous to the Stokes drag factor 6*πμR* for a sphere of radius *R*, via the relation$\partial F/\partial \left({\dot{\gamma }}_{e}\ell \right)=-\lambda_{e}\mu \ell$, with *λ*_*e*_ = *αF*(0) / *μ𝓁V* (0). For a bundle applying a force *F*_0_ in an otherwise quiescent fluid, we infer an equivalent response *F* − *F*_0_ to a general shear rate $\dot{\gamma }$, and drag coefficient $\partial F/\partial (\dot{\gamma }\ell )=-\lambda \mu \ell$. Bundles experience the Sum $\dot{\gamma }={\dot{\gamma }}_{e}+{\dot{\gamma }}_{a}(F)$, where ${\dot{\gamma }}_{a}(F)=\partial u_{a}/{\partial z|}_{z=0}$. The constant *λ* can be related to the empirical parameter *λ*_*e*_ by matching derivatives of *F* − *F* (0) and *F* − *F*_0_ with respect to ${\dot{\gamma }}_{e}$. As *d*/ Λ ≪ 1, we have ${\dot{\gamma }}_{a}\approx 3F\ell /\mu d^{3}$, and thus *λ =* [1/*λ*_*e*_ − 3 (𝓁* / d*)^3^]^−1^ ≈ 8.6 ± 2.5 for *Xenopus*; this is within a factor of 2 of the value 6*π* for a sphere.

To obtain the self-consistent model, we set ${\dot{\gamma }}_{e}=0$, and find $F=F_{0}-\mu \lambda \ell^{2}{\dot{\gamma }}_{a}(F,d)$. For *ϕ* = (𝓁* / d*)^2^ ≪ 1 we find

(3) $$F(d)\approx \frac{F_{0}}{1+3\lambda {\left(\ell /d\right)}^{3}},$$

so the force applied by a bundle decreases with coverage fraction *ϕ*, as does the derivative ∂*U* / ∂*ϕ* of the limit velocity *U* ∝ *Fϕ* in the outer region (2). With *U* bounded by the cilia tip velocity *V*, the observation that *U* ~ *V* / 2 at *ϕ <* 0.1, is indeed consistent with lower rates ∂*U* / ∂*ϕ* at larger *ϕ*. This implies a trade-off between the dissipated force *F*_0_ − *F*, and the benefits of the collective contribution *u*_*a*_, as seen in the extra shear force per bundle $F_{w}\approx \mu {\dot{\gamma }}_{a}\left(d^{2}-\ell^{2}\right)$ applied to non-ciliated cells and the extra force per bundle *F*_*a*_ *= F*𝓁* / d*. The latter relates to the power $FU=F_{a}^{2}/\mu 𝓁$ contributing to long-range transport [29]. Periciliary and outer transport are coupled for sparse tissues, as$F_{w}^{2}\;\sim {\left(\mu {\dot{\gamma }}_{a}d^{2}\right)}^{2}=9\mu \ell FU$. The latter is maximal at *d*_max_ = (6*λ*)^1/3^𝓁 ≈ 56 *μ*m, suggesting that further increase in coverage is inefficient.

In Fig. 4, we estimate these forces for all *ϕ* using the exact value of ${\dot{\gamma }}_{a}$ for a distribution of point forces [(S8,9), [29] ]. The optimum of *F*_*w*_ sharply decreases with *λ* and for *Xenopus* is at *ϕ* ~ 0.055, very close to the *in vivo* value. Increasing *ϕ* primarily increases transport in the outer region, where the collective power *FU* is maximal at *ϕ* ~ 0.1. At *λ* = 0, *F*_*w*_ is maximal at *ϕ* = 0.17, primarily because the nonciliated area goes to zero with *ϕ*, while *FU* is maximal at *ϕ =* 1. The observed mosaic patterns are nearly optimal based on the clearing force *F*_*w*_.

Similar results arise from the envelope approach to flows driven by arrays of finite-size bundles [29]. For *ϕ >* 0.33 these models lose realism as the vortices of size ~2*𝓁* start to overlap, but we expect an optimum shearing configuration at *ϕ <* 0.3, as transport will be confined to the region above the cilia for larger *ϕ*. Similar dissipative phenomena will contribute to systems working at larger coverage fractions, as in airways [14], though synchrony of bundles as well as mucus between cilia and the air above add complexity.

We close with connections to other sparse distributions of active elements. The force applied to the fluid by the cilia tips on the envelope ∂Ω_*c*_ is equal and opposite to that applied to the skin, and we can simplify our results on the shearing of nonciliated cells by reconsidering the flow *u*(*z*) = *UG*(*z*/ Λ) in Eq. (2) above a patch of activity of radius Λ with slip velocity *U*. The shear stress driving the flow is *τ*_Λ_ ≈ −3*μU* / 2Λ, which we assume constant over a bundle. Setting Λ = 𝓁 and integrating over a tissue with *N* bundles we obtain *J* ~ *N*𝓁^2^*τ*𝓁. By contrast, if we set Λ = *R*, the local shear stress is *τ*_*R*_ *=* − 3*μU* / 2*R* and the force over the entire surface is *J*_*R*_ ~ *πR*^2^*τ*_*R*_. With *N = π R*^2^ *ϕ* / 𝓁^2^, the ratio

(4) $$\frac{J}{J_{R}}\sim \frac{R}{\ell }\phi$$

measures how a distribution of noninteracting MCCs shears the surface relative to the collection. The linear scaling with *ϕ* is expected, but the large prefactor *R* / 𝓁 ~ 20 (system size/MCC size) implies that *J / J*_*R*_ can approach unity for *ϕ* as low as ~ 𝓁* / R* ~ 5%. This mirrors Jeffreys’ result [42] for the evaporation rate from sparse leaf stomata, rediscovered years later [43] in the context of ligand binding to cell receptors [44].

The results presented here show that long-range hydrodynamic interactions between multiciliated cells allow efficient periciliary transport at low coverage, favoring coexistence of multiple cell types. This is likely but one example of the mechanisms that maintain efficient transport in the upscaling events marking evolutionary transitions from unicellular to larger multicellular systems.

## Supplementary Material

Boselli_etal_SM

Boselli_etal_SupMovie1

## Figures and Tables

**Fig. 1:**
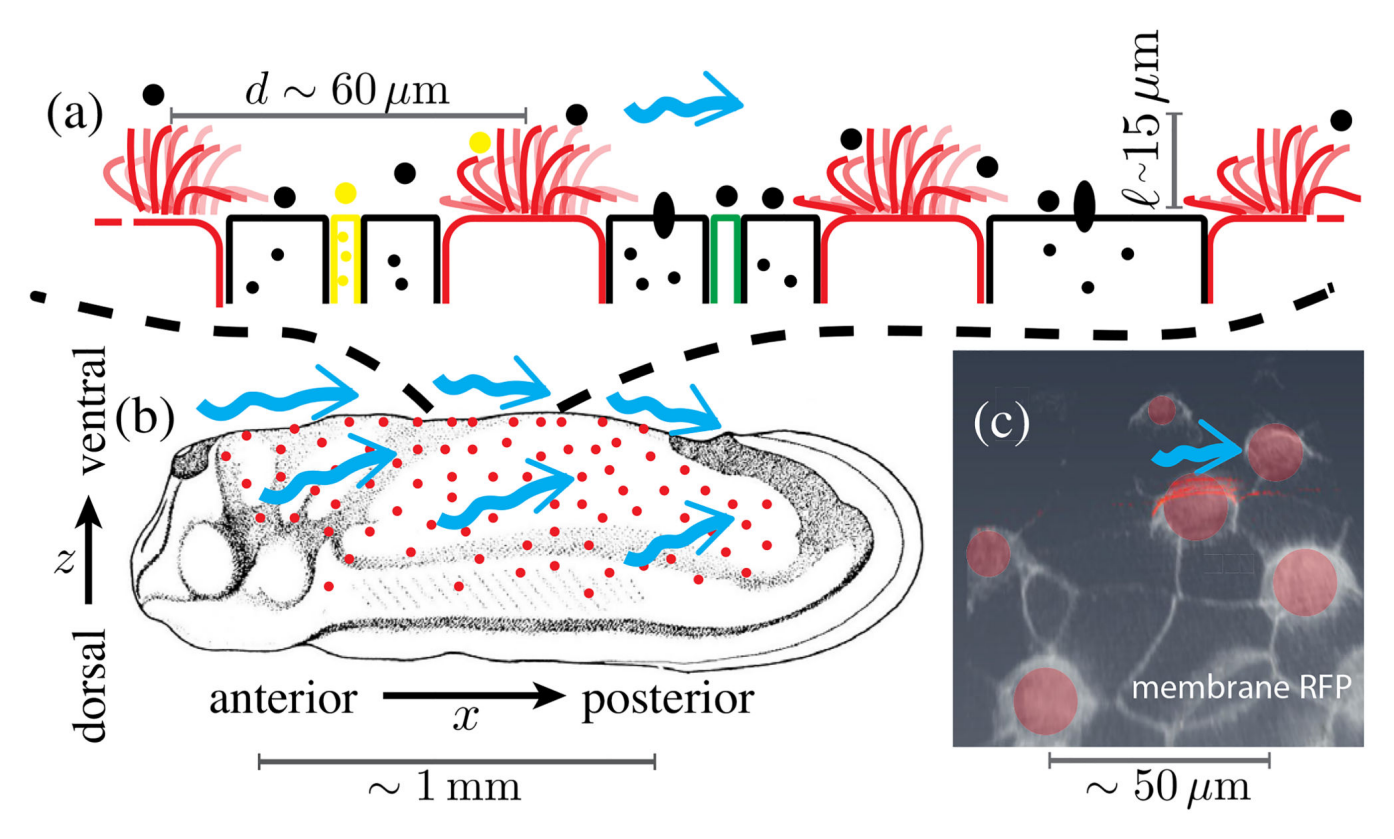
Ectoderm of embryonic *Xenopus laevis* at tailbud stages. (a) Schematic side view of MCCs (red) intermixed with secreting cells. (b) Location of MCCs across the embryo (adapted from Refs. [22,23]) and cilia-driven flow (blue arrows). (c) Con-focal image of cell membranes (stained by membrane-RFP), with MCCs segmented in red, in ventral region of skin.

**Fig. 2:**
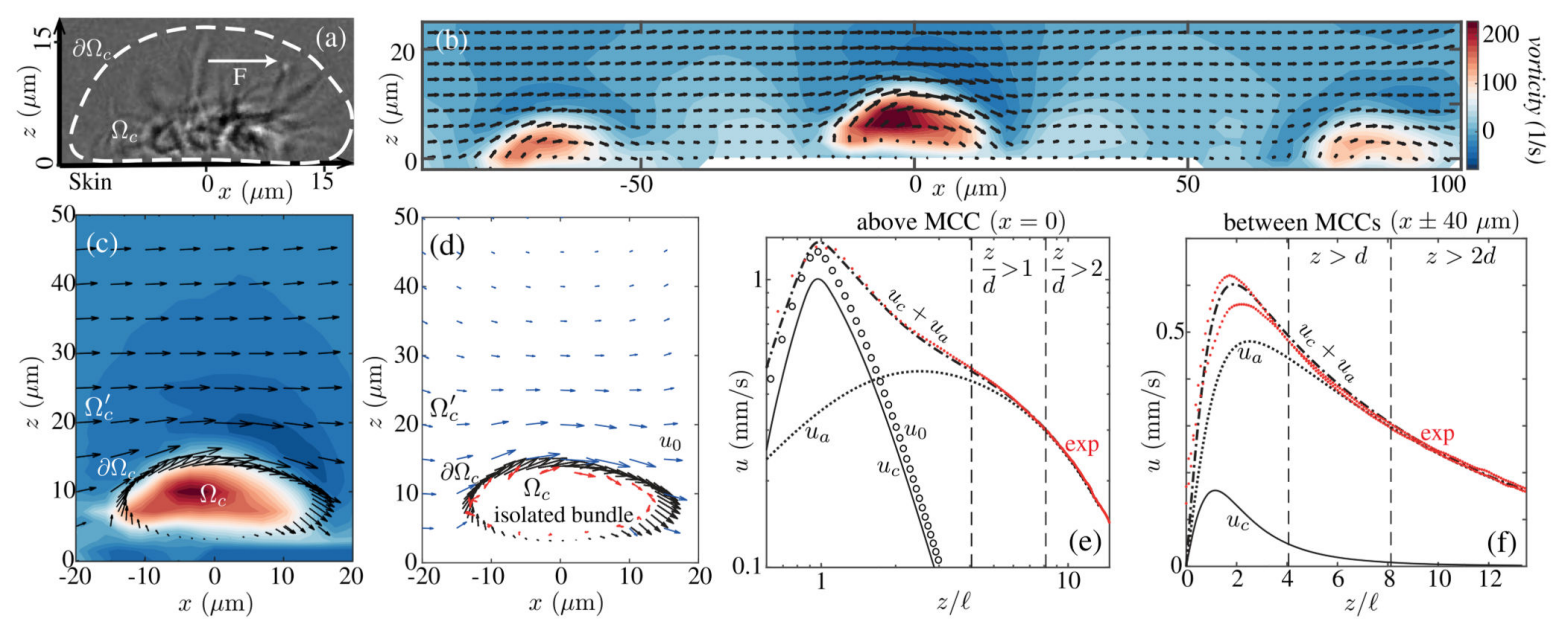
Flow fields. (a) Lateral view of MCC showing (dashed) path of cilia tips and force **F**. (b) Experimental velocity field and vorticity in plane normal to skin near several MCCs. (c) Near an MCC, as in (b), with direction of cilia tip motion (black arrows) on ∂Ω_*c*_.(d) Estimated flow field *u*_0_ for an isolated MCC (blue arrows): Point forces (red arrows) are used to fit velocity near cilia tips. Lateral velocity at (e) *x, y* (0, 0) and (f) (±40 *μ*m, 0) in experiment (exp) and theory, with *u*_0_ driven by an isolated bundle and *u*_*c*_ by a bundle exposed to endogenous flow *u*_*a*_ (see also Figs. S2–S4 [29]).

**Fig. 3:**
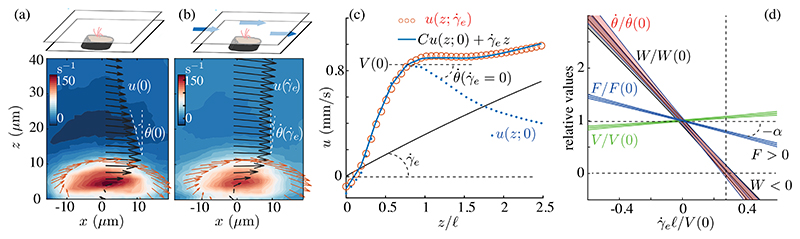
Response of cilia bundles to exogenous shear. (a),(b) Vorticity and velocity vectors before and during perfusion. (c) Velocities *u*(*z*; 0) and $u\left(z;{\dot{\gamma }}_{e}\right)$, shear flow ${\dot{\gamma }}_{e}z$, and sum $Cu(z;0)+{\dot{\gamma }}_{e}z$ fitting $u\left(z;{\dot{\gamma }}_{e}\right)$, where $C=F\left({\dot{\gamma }}_{e}\right)/F(0)$. (d) Variation with shear of estimated force *F*, velocity *V* and shear rate $\dot{\theta }=\partial u(z)/{\partial z|}_{\ell }$ measured above cilia tips, and rate of work $W\propto \dot{\theta }V$ (overlapping $\dot{\theta }$), normalized by values at ${\dot{\gamma }}_{e}=0$. Shaded regions are 95% confidence intervals of averages over 10 samples.

**Fig. 4:**
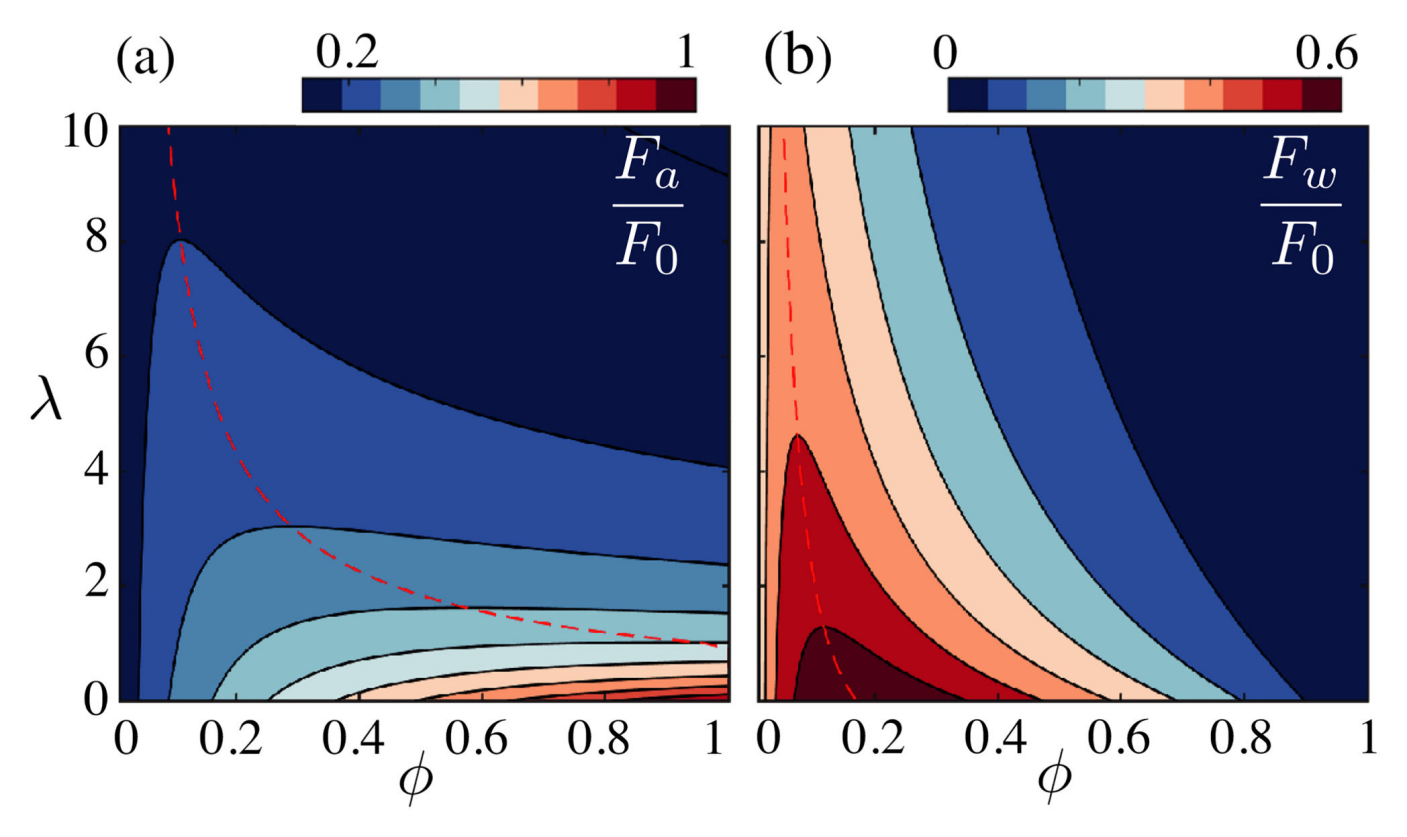
Collective efficiency of a distribution of point forces, in the space of coverage *ϕ* and dissipative coupling constant *λ*. Dashed lines trace optimization ridges of the extra forces per bundle *F*_*a*_ and *F*_*w*_ driving (a) flow in the outer region and (b) shear above nonciliated cells.
