# Supplementary material for: Fluid Mechanics of Mosaic Ciliated Tissues
Source: Phys Rev Lett. Author manuscript; Available in PMC 2024 Jun 11. (PMC7616087; doi:10.1103/PhysRevLett.127.198102)
Supplement: Boselli_etal_SM [file EMS196595-supplement-Boselli_etal_SM.pdf]

Supplemental Material:
Fluid mechanics of mosaic ciliated tissues

Francesco Boselli,[1,*] Jerome Jullien,[2,3,4,†] Eric Lauga,[1,‡] and Raymond E. Goldstein[1,§]

[1] *Department of Applied Mathematics and Theoretical Physics, Centre for Mathematical Sciences, University of Cambridge, Cambridge CB3 0WA, United Kingdom*

[2] *Wellcome Trust/Cancer Research UK Gurdon Institute, Tennis Court Road, Cambridge CB2 1QN, United Kingdom*

[3] *Department of Zoology, University of Cambridge, Cambridge CB2 1QN, United Kingdom*

[4] *Inserm, Nantes Université, CHU Nantes, CRTI-UMR 1064, F-44000 Nantes, France*

(Dated: October 26, 2021)

This file contains additional experimental and calculational details.

Embryo Culture. *Xenopus* embryos were prepared as described previously [S1]. Briefly, mature *Xenopus laevis* males and females were obtained from Nasco [S2]. Females were injected with 50 units of pregnant mare serum gonadotropin 3 days in advance and 500 units human chorionic gonadotropin 1 day in advance in the dorsal lymph sack to induce natural ovulation. Eggs were laid in a 1× MMR buffer (5 mM HEPES pH 7.8, 100 mM NaCl, 2 mM KCl, 1 mM $MgSO_4$, 2 mM $CaCl_2$, 0.1 mM EDTA). *Xenopus* embryos were cultured at room temperature or 15° C in the 0.1× MMR until they reached stage 27/28. Experiments with embryos were performed at the late tailbud stages (stages 28-30, as describe in Faber and Nieuwkoop [S3]). Embryos were terminated humanely immediately following the experiments.

Our work with *Xenopus laevis* is covered under the Home Office Project License PPL 70/8591 and frog husbandry and all experiments were performed according to the relevant regulatory standard. All experimental procedures involving animals were carried out in accordance with the UK Animals (Scientific Procedures) Act 1986. Moreover, we only used surplus embryos for this study, to conform with the NC3Rs guidance to exploit the possibility to minimise the use of animals by sharing embryos with collaborators.

Pulsatility of the flow field. Since the cilia within a bundle do not move in phase, and the far field contribution arises from many bundles that are also not in strict phase coherence, the pulsatility of the flow is low compared to the time average $\bar{u}$. Examples of the velocity signal $u(t)$ above and between bundles (cf. Fig. 2b) are shown in Fig. S1. The relative root mean square value of the fluctuations $(u(t)-\bar{u})/\bar{u}$ is $\sim$0.2 at the cilia tips, and it decays to $\sim$0.1 for $z > 2\ell$, as well as between bundles, on top of non ciliated cells. The steady component of the flow is then the most relevant, and in this study we consider only $u = \bar{u}$. This does not exclude more intricate synchronization phenomena between bundles.

Statistics. To fit any quantity y measured at a given hydrodynamic load x, we assume a linear relation $y = a + bx$ and find 95% confidence intervals for the averages $\bar{a}$ and $\bar{b}$ of the parameters a_i and b_i given by a least-square fit of the measured values $(x_{i,m}, y_{i,m})$ acquired for the i-th MCC. We have [S4]:

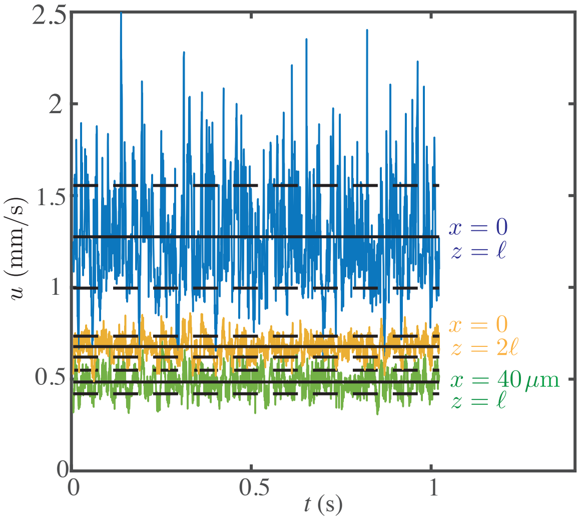

FIG. S1. Lateral velocity $u(t)$ over time (raw PIV-data) sampled at a point at the cilia tips (blue; $x = 0$, $z \approx \ell \sim 15\,\mu$m); above the cilia (yellow; $x = 0$, $z \approx 2\ell$); between two bundles (green; $x = 40\,\mu$m, $z = \ell$). Coordinates are those of Fig. 2(b). Black solid lines: time averages; Dashed lines: r.m.s. of the fluctuations around their time average.

$$\bar{a} = \frac{\sum_{i=1}^{N} a_i/\sigma_{a,i}^2}{\sum_{i=1}^{N}(1/\sigma_{a,i}^2)} \pm 2\sigma_{\mu\bar{a}} \tag{S1a}$$

$$\bar{b} = \frac{\sum_{i=1}^{N} b_i/\sigma_{b,i}^2}{\sum_{i=1}^{N}(1/\sigma_{b,i}^2)} \pm 2\sigma_{\mu\bar{b}}, \tag{S1b}$$

The standard errors for the mean parameters are the square roots of

$$\sigma_{\mu\bar{a}}^2 = \frac{1}{\sum_{i=1}^{N} 1/\sigma_{a,i}^2} \tag{S2a}$$

$$\sigma_{\mu\bar{b}}^2 = \frac{1}{\sum_{i=1}^{N} 1/\sigma_{b,i}^2}, \tag{S2b}$$

while the parameter variances for the i-th MCC are

$$\sigma_{a,i}^2 = \frac{\sigma_i^2}{\triangle_i'} \sum_{m=1}^{M} x_{i,m}^2 \quad \text{(S3a)}$$

$$\sigma_{b,i}^2 = M \frac{\sigma_i^2}{\triangle_i'}, \quad \text{(S3b)}$$

with

$$\triangle_i' = M \sum_{m=1}^{M} x_{i,m}^2 - \left(\sum_{m=1}^{M} x_{i,m} \right)^2 . \quad \text{(S4)}$$

The prime superscript indicates that the variance of the measurements $\sigma_{i,m}^2 = \sigma_i^2$ for the i-th MCC was assumed to be constant. It was estimated as the variance s^2 of the sample population:

$$\sigma_i \approx s^2 = \frac{1}{N-2} \sum_{m=1}^{M} (y_m - a_i - b_i x_m)^2. \quad \text{(S5)}$$

We normally imaged $M = 4$ conditions per MCC. For exceptions with $M = 2$, s^2 could not be computed directly from (S5) and was assumed to equal the largest value from the other experiments.

Confocal imaging and cell density To estimate density in the ventral region of the same embryos where we performed PIV measurements we proceeded as follows. Bright field images, as presented in the main text, allow us to estimate the distance $d_{11} \approx 70 \pm 15\,\mu$m between bundles in the same focal plane, that is, along the streamflow direction. As the high-speed camera used for the PIV analysis discussed in the main text was mounted on a Zeiss CellObserver SD spinning disk system, we could locate bundles along the y-direction by sequentially performing confocal imaging of the fluorescent beads used for the PIV analysis. Particle traces from overexposed images were obtained by setting the exposure time to 20 ms. These appear as straight segments outside the bundle, while bundles can be identified as vortical structures. The distance d_{22} between bundles along the y-direction was then estimated by stacks of images acquired at y positions spaced apart by $2\,\mu$m. Density was then estimated as $\mathcal{P} = d_{11} d_{22}$. For the embryo which data are illustrated in Fig. 2 we obtain $d_{22} \approx 54.3 \pm 8\,\mu$m, and $\mathcal{P} \approx 2.6 \pm 0.5 \times 10^{-4}\,\mu\text{m}^{-2}$.

To estimate the density of multiciliated cells, we also analyzed confocal images of embryos injected at one-cell stage with membrane-RFP (marking cell membrane), at times co-injected with CLAMP-GFP (marking the rootlets of the basal bodies) [S5]. Plasmids expressing Clamp were kind gifts from Dr John Wallingford [S6]. This analysis was mostly performed for regions in the lateral side of the embryos, which is closer to the objective and more suitable for confocal imaging. We obtain $\mathcal{P} \approx 2.75 \pm 0.7 \times 10^{-4}\mu\text{m}^{-2}$, with ± 0.7 the standard deviation between experiments. The typical cell area was also estimated from those images.

Fitting the near flow-field by the singularity method. The flow u_c driven by the cilia in Ω_c is modelled as the superposition of the flows arising from local point forces (Stokeslets) $\mathbf{f}_n$ applied at $\mathbf{s}_n \in \Omega_c$. The values of $\mathbf{f}_n$ are found by fitting $\mathbf{u}_c$ at M collocation points $\mathbf{b}_i$, solving the linear system

$$\mathbf{u}_c(\mathbf{b}_i) = \sum_{n=1}^{N} \mathbf{f}_n \cdot \mathbf{S}(\mathbf{b}_i, \mathbf{s}_n). \quad \text{(S6)}$$

The Blake tensor $\mathbf{S}$ is the well-known, exact solution for a point force (or Stokelset) next to a no-slip plane at $z = 0$ [S7]. As no-slip boundary conditions $\mathbf{u}_c = \mathbf{0}$ at $z = 0$ are implicitly satisfied, walls do not need to be discretized. The linear system (S6) is then simply recast in its matrix form $\mathbf{A}\mathbf{f} = \mathbf{u}_b$, with the $3M \times 2N$ matrix

$$\mathbf{A} = \begin{pmatrix} S_{xx}(\mathbf{b}_i, \mathbf{s}_j) & S_{xz}(\mathbf{b}_i, \mathbf{s}_j) \\ S_{yx}(\mathbf{b}_i, \mathbf{s}_j) & S_{yz}(\mathbf{b}_i, \mathbf{s}_j) \\ S_{zx}(\mathbf{b}_i, \mathbf{s}_j) & S_{zz}(\mathbf{b}_i, \mathbf{s}_j) \end{pmatrix}$$

the $2N \times 1$ vector $\mathbf{f} = \{f_{1,x}, \ldots, f_{N,x}, f_{1,z}, \ldots, f_{N,z}\}$, and the $3M \times 1$ vector $\mathbf{u}_b = \{u_{c,1}(\mathbf{b}_i), u_{c,2}(\mathbf{b}_i), u_{c,3}(\mathbf{b}_i)\}$. We set $f_{n,y} = 0$, assuming the solution to be symmetric in y. To avoid numerical instabilities, we take $M > 2N$ [S8], and then solve for $\mathbf{f}$ using the backslash operator of Matlab. Once the $\mathbf{f}_n$ are known, (S6) can be used to evaluate the fitted solution not only at $\mathbf{x} = \mathbf{b}_i$, but at any position $\mathbf{x}$.

Similar solutions can be obtained for different sets of source and collocation points. For the results shown, the flow field measured in the y plane was extruded by replications at 13 planes evenly spaced between $-10\,\mu\text{m} < y < 10\,\mu\text{m}$. We used 15 Stokeslets for each plane about the fictitious boundary $\partial\Omega_c$.

To account for uncertainties related to the location of collocation and source points, fittings were repeated for several configurations. For a given set of collocation and source points, the uncertainty in the effective force F is estimated as $\sigma_F = F||\mathbf{A}\mathbf{f} - \mathbf{u}_b||_{L2}/||\mathbf{u}_b||_{L2}$. This calculation was repeated for each of the T configurations tested. The i-th configuration gives the effective force F_i and uncertainty $\sigma_{F,i}$. Then we calculate the weighted average $F = \sum_{i=1}^{T} F_i \sigma_{F,i}^2 / \sum_{i=1}^{T} \sigma_{F,i}^2$, and variance $\sigma_F^2 = T / \sum_{i=1}^{T} \sigma_{F,i}^{-2}$. In a similar way, the propagation of uncertainties about d, when fitting $u - u_a(d)$, are estimated by repeating the analysis for several values of d within the range $d \pm \sigma_d$, and averaging as above. Uncertainties in the main text are given as $F \pm \sigma_F$.

Coarse-graining the bundle. The flow $\mathbf{u}_c$ driven by the forces in the bundle can be coarse-grained further, with a smaller number of Stokes flow singularities, moving away from the bundle. We compare the fitted

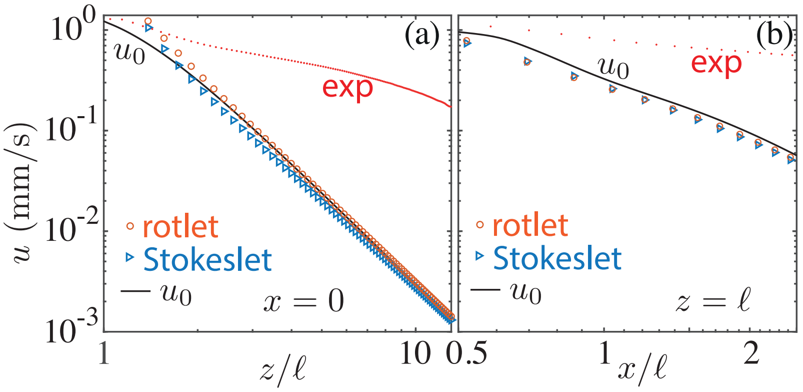

FIG. S2. Comparison of experimental and theoretical flow profiles near an MCC. (a) Lateral velocity u_0, with reference to Fig. 2(d), above the bundle as a function of z, and (b) between bundles at $z = \ell$, as a function of x. The flow arising from a single bundle of cilia decays as that of an effective force F (Stokeslet) or moment $2\ell F$ (rotlet), while the measured flow profile (exp) decays much more slowly due to the contributions from other MCCs.

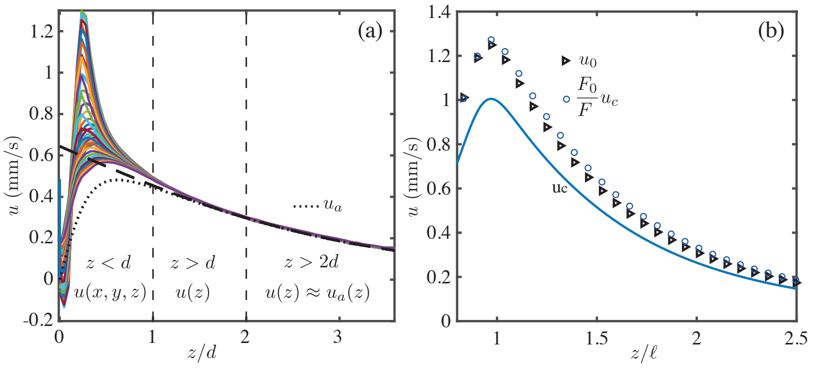

FIG. S3. Supplement to Fig. 2 of main text. (a) Measured lateral velocity $u(z)$ above and between bundles. For $z > d$, $u(z)$ becomes independent of x, while for $z > 2d$, it matches the ambient flow $u_a = U[G(z/\Lambda) - G(2z/d)]$ arising from a uniform distribution of point forces. The dashed line is the asymptotic solution $u_a \approx UG(z/\Lambda)$ for large values of z. Different colours correspond to velocity profiles at various values of $|x| < 35$ μm. (b) Linear dependence of the near-field $u_c(z)$ above a bundle ($x = 0$) on the effective force F, for $z > \ell$. The numerical solution $u_0(z)$ corresponding to the effective force F_0, can be approximated by re-scaling the solution $u_c(z)$ by a factor F_0/F.

flow $\mathbf{u}_c$, made up of N point forces as discussed above, with the flow driven by the effective force $F\mathbf{e}_x$ applied at $(0, 0, \ell)$. The flow driven by the entire bundle decays as $1/z^3$, as for a single singularity, for $z > 2\ell$ (Fig. S2). Results are shown for the velocity component u_0 with reference to Fig. 2, and $u_s = F_0 S_{xx}$.

Very similar results arise if the flow is driven by an effective torque (giving a rotlet) $2\ell F\mathbf{e}_y$ applied at $(0, 0, \ell/2)$ next to a no-slip wall. The fact that the near field of a point torque is a swirling flow, but the far-field flow $\sim zx^2/(\rho^2 + z^2)$ is the same as for a point force, reflects what was discussed for the cilia driven flow u_c, which indeed appears as an active vortex within the bundle, but decays as a point force in the far field.

Far Field fitting. The flow u_0 gives a first approximation of the flow velocity driven by a single bundle. As discussed above and in the main text, it decreases much faster than the measured velocity $u(z)$. For $z > 2d$, we have $u_0/u < 0.02$ and $G(2z/d)/G(z/\Lambda) < 0.003$, such that in the far-field $u \approx UG(z/\Lambda)$. The parameters Λ and U can then be obtained by using u_a to fit the PIV measurements for $z > 2d$ (Fig. S3).

The velocity $u_a(z)$ depends linearly on U, but non-linearly on Λ. For a given value of Λ, we find $U(\Lambda)$ by a linear least-squares fit of the data. We then repeat this linear fit for candidate values in the range $30\,\mu$m $< \Lambda < 1$ mm, with increment $\Delta\Lambda = 1\,\mu$m, and select the value of Λ that minimize the error $\sigma = \sum_{m=1}^{M}(VG(z/\Lambda) - u)^2/(M-1)$ at M collocation points. Relative fitting errors remain below 0.005%. Uncertainties σ_Λ and σ_U of the best fitting parameters Λ and U are estimated from the relationships $\sigma^2 = f(\Lambda + \sigma_\Lambda)^2$,

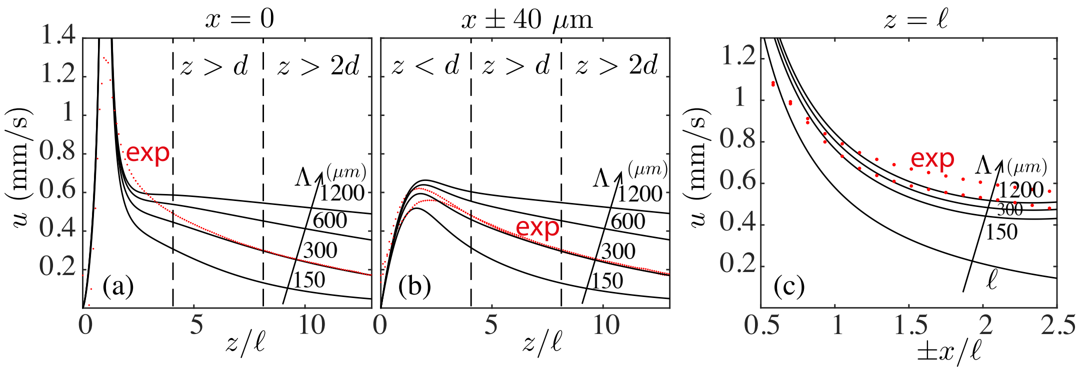

FIG. S4. The lateral velocity of a two-dimensional array of Stokeslets of radius $\Lambda \sim 300$, falls off as in experiments (exp), as shown for: (a,b) above and between bundles, respectively, as a function of z; (c) between bundles as a function of x. Results are obtained by direct summation of the exact solution of each point force, all of strength $F\mathbf{e}_x$ and z-offset ℓ.

and $\sigma_U^2 = f(\Lambda + \sigma_\Lambda)^2$, which are computed numerically.

Two-dimensional array of Stokeslets. Results similar to those presented in Fig. 2 for a uniform distribution of forces can be obtained by positioning point forces $F\mathbf{e}_x$ on a lattice with cut-off radius Λ. Each element $\mathbf{s}_{ij}$ of the lattice is positioned at $(x_{ij} = id_{11} + jd_{12}, y_{ij} = jd_{22}, z_{ij} = \ell)$. From confocal imaging of the closest neighboring cells of the bundle in Fig. 2, we estimate $d_{11} \sim 70\,\mu$m, $d_{12} = 40.5\,\mu$m, $d_{22} = 53\,\mu$m.

Using the effective force F estimated by the near field fitting, and summing up the exact contribution of each MCC, we retrieve the slow decay rate observed *in vivo* [Figs. 2(e,f)] for $\Lambda \sim 300\,\mu$m (Fig. S4). This is the same result found by fitting the far-field flow with Eq. (2).

Resistive force theory estimate of the effective force applied by a single cilium. We adopt a simplified view of the power stroke of a cilium as a straight rod that pivots around its base. Let $s \in [0, \ell]$ be arclength along a cilium, with $s = 0$ at the base and $s = \ell$ at the tip, and let ϕ be the angle between the cilium and the wall. The lateral component of the RFT force density at s is $f' \sim (s/\ell)\zeta_\perp V_c \sin\phi$, and the resulting far field velocity, given by Eq. (1), is proportional to $hf'ds$ with $h = s\sin\phi$. Accordingly, the effective force $f_\phi = \ell^{-1}\int_0^\ell h(s,\phi) f'(s,\phi) ds$, matches the overall far field $f_\phi \tilde{S}_{xx}$ when applied at ℓ. We obtain $f_\phi = \sin^2\phi\, \zeta_\perp \ell V_c/3$. Through the entire stroke, a cilium cycles through an angle $\Delta\phi = 2\pi$, and we assume that the recovery stroke does not contribute to the force, so $f_\phi = 0$ for $\pi < \theta < 2\pi$. Averaging f_ϕ gives the effective force $f = \zeta_\perp \ell V_c (6\pi)^{-1} \int_0^\pi \sin^2\phi\, d\phi$, which gives the expression $f = \zeta_\perp \ell V_c/12$ used in the main text.

Additional results from self-consistent model. The self-consistent model in Eq. (5) [Fig. S5(a)] can be used to investigate several aspects of the phenomenology of sparse cilia driven flow. To improve the near-field approximations at larger values of $\phi = (\ell/d)^2$, we find the exact shear rate $\dot\gamma_a$ driven by a uniform distribution of point forces $F\mathbf{e}_x$ applied at $z = \ell$, with density $\mathcal{P} = 1/d^2$:

$$\dot\gamma_a(F, \mathcal{P}) = \mathcal{P}F \int_0^{2\pi} \int_{d/2}^{\infty} \rho\, d\rho\, d\theta\, \frac{\partial S_{xx}}{\partial z}(\rho, \theta, z = 0), \quad \text{(S7)}$$

where we consider the exact Blake tensor S_{xx} rather than its far field approximation. We obtain

$$\dot\gamma_a(F, d) = \frac{F\ell}{2\mu d^2} \frac{2\ell^2 + 3d^2/4}{(\ell^2 + d^2/4)^{3/2}}, \quad \text{(S8)}$$

and the relationship between F and F_0 becomes:

$$F = F_0 \left[1 + \lambda \frac{\ell^3}{2d^2} \frac{2\ell^2 + 3d^2/4}{(\ell^2 + d^2/4)^{3/2}}\right]^{-1}, \quad \text{(S9)}$$

which for large d tends to (3) (Fig. S5). The forces F_w and F_a computed using (S8,9) are shown in Fig. 4 of the main text.

Additional comments on the efficiency of mosaic ciliated tissues. Here we elucidate the relation

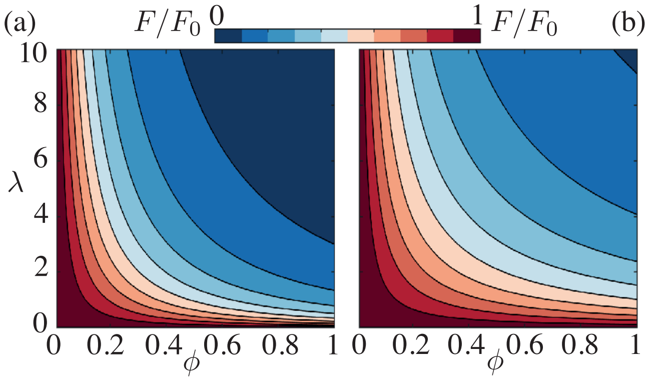

FIG. S5. Supplement of Fig. 4. Contour plot of F/F_0 for (a) the asymptotic result [Eq. (3)], and (b) the more accurate solution [Eq. (S9)].

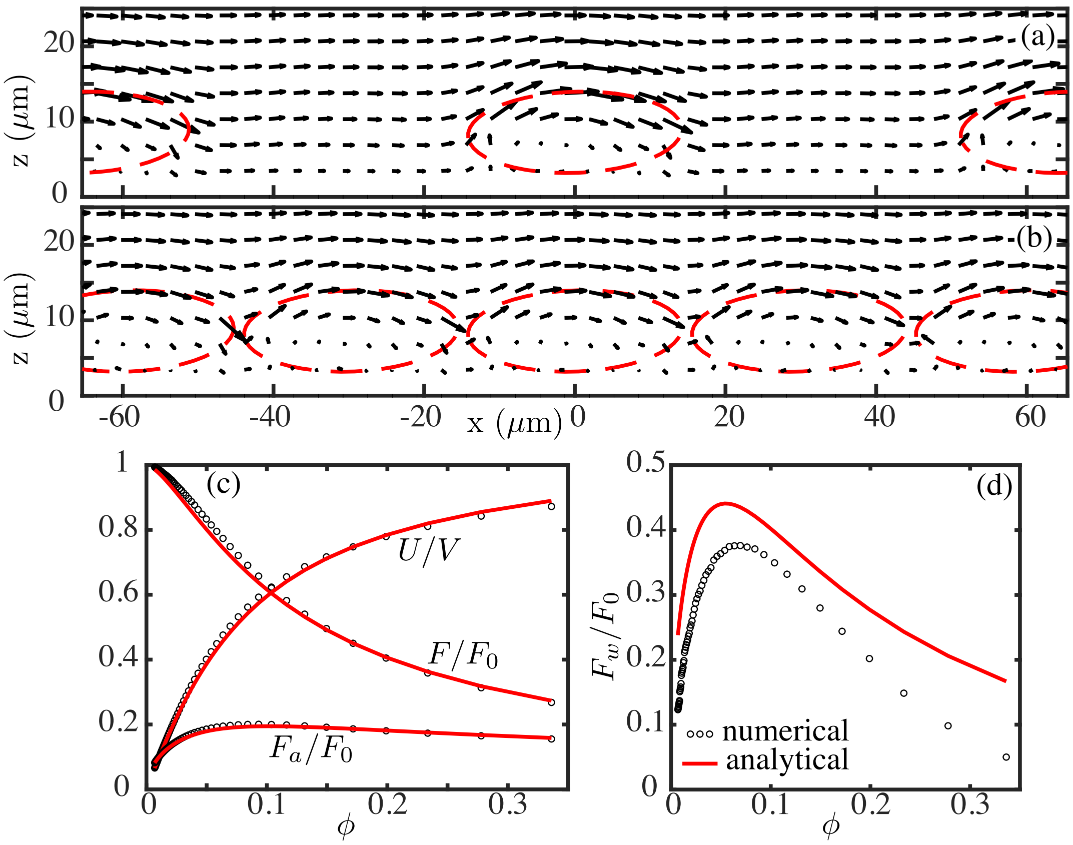

FIG. S6. Computational model of arrays of finite-size bundles. (a) and (b): lateral views of the velocity field (vectors) predicted at coverage fractions $\phi \approx 0.07$ and $\phi \approx 0.34$, respectively; dashed lines indicate the fictitious boundaries $\partial\Omega_c$ of the vortexes driven by the bundles, where velocity is prescribed. (c) Effective force F, limit velocity $U \propto F/d^2$, extra pumping force per bundle $F_a = \sqrt{FV\mu\ell}$, and (d) extra shearing force per bundle F_w as a function of the coverage fraction (non dimensional density) ϕ. Forces are normalized by the effective force F_0 computed at $\phi = 0$. The velocity U is normalized by the cilia tip velocity V, taken as the maximum velocity magnitude prescribed at $\partial\Omega_c$. Results of numerical simulations (circles) for finite-size bundles are compared with analytic results (solid lines) for the point force distribution discussed in the main text, with F given by (S9) and $\lambda = 8.6$.

between the metrics for the efficiency of mosaic patterns discussed in the main text and the collective efficiency of cilia carpets $\epsilon = \mu\mathcal{P}Q^2/\ell E$ introduced elsewhere [S9], where $Q = \int_{x=0} u\, dA$ is the volume flow rate through any zy-plane and E is the energy dissipated by the cilium. The goal in that earlier work was to optimize the cilia waveform.

Recalling that $U = \pi Q/d^2$ [S9], we also have $\epsilon = FU/\pi E$. Assuming E is the constant total energy uptake by the MCC, FU gives the pattern efficiency as the contribution per bundle to the power generated in the outer region. In the far field limit, $FU \approx F_w^2/9\mu\ell$, with $F_w \approx \mu\dot{\gamma}_a d^2 = 3F\ell/d$ the collective contribution to force applied to the wall.

For large coverage fractions, the far field approximation of $\dot{\gamma}_a$ becomes inaccurate, and the finite size of the bundle becomes important, and the shearing efficiency F_w^2 of the pattern decreases faster than its pumping efficiency FU with ϕ. Therefore, we define the extra shearing force as $F_w = \mu\dot{\gamma}_a(d^2 - \ell^2)$, and use the exact formulation of $\dot{\gamma}_a$ (S8) to improve its near field approximation. For comparison, we find useful to define the extra force $F_a = \sqrt{\mu\ell FU}$, which we interpret as the extra force per bundle driving fluid transport in the outer region.

Two-dimensional array of finite-size bundles. Fig. S4(c) shows how the approximation of bundles through single point forces can quite conveniently capture the lateral flow velocity measured between bundles, up to their proximity. Yet, at large coverage fractions, this approximation may become inaccurate. To support our results for a uniform distribution of point forces (Fig. 4), we report here results from a numerical study of finite-size bundles of prescribed kinematics positioned on a lattice. We build on (S6) and the configuration presented in Fig. 2(d): Each bundle ξ is modelled by N point forces $\mathbf{f}_n$ applied at $\mathbf{s}_{\xi,n}$ within the bundle domain $\Omega_{c,\xi}$, which are determined by enforcing kinematics measured about the trajectory of the cilia tips.

The lattice is regular and all the bundles identical, such that the corresponding forces $\mathbf{f}_n$ and prescribed velocities are the same in any bundle. For simplicity, we take as reference the bundle at the origin $\mathbf{c}_0 = (0, 0, 0)$. The corresponding point forces are applied at $\mathbf{s}_{0,n}$. For the other bundles, centered at $\mathbf{c}_\xi = (x_\xi, y_\xi, 0)$, the corresponding forces are applied at $\mathbf{s}_{\xi,n} = \mathbf{c}_\xi + \mathbf{s}_{0,n}$. The flow driven by such an array of in-silico bundles is

$$\mathbf{u}(\mathbf{x}) = \sum_{n=1}^{N} \mathbf{f}_n \cdot \sum_{\xi=0}^{\Xi-1} \mathbf{S}(\mathbf{x}, \mathbf{s}_{\xi,n}). \tag{S10}$$

We find $\mathbf{f}_n$ by fitting $\mathbf{u}(\mathbf{b}_i)$ to the velocity measured at M collocation points $\mathbf{b}_i$ at the fictitious boundary $\partial\Omega_c$ of a reference bundle. The resulting linear system is similar to (S6), but summed over Ξ bundles as in (S10).

The positions of the bundles are given by the lattice positions $(x_{ij} = id_{11} + jd_{12}, y_{ij} = jd_{22})$. We present results for the typically observed ratios $d_{22}/d_{11} \approx 0.76$ and $d_{12}/d_{11} \approx 0.58$. We keep the cut-off radius $\Lambda = 300\,\mu$m as found previously. Computations were repeated for various values of d_{11}, corresponding to a coverage fraction $\phi = \ell^2/d_{11}d_{22}$. We tested other lattice configurations and they did not change the observed phenomenology.

The effective force F of each bundle decreases with ϕ. The comparison with predictions from (S9) for a point force distribution and $\lambda = 8.6$ is excellent, as well as for the force density and related limit velocity $U \propto F\phi$, and the extra force per bundle $F_a = \sqrt{FU\mu\ell}$. The extra shearing force per bundle F_w applied to non-ciliated tissue by u_a is computed numerically. We compute the ambient flow as $u_a(x, y) = u - u_c$, where u_c is the contribution of the bundle at the origin, and then integrate $\dot\gamma_a(x, y) = \partial u_a(x, y, z)/\partial z|_{z=0}$ on the domain $(\max b_{x,i} < |x| < d_{11}, \max b_{y,i} < |y| < d_{22})$, which excludes the area below the volume spanned by the cilia bundle. For the point force distribution, we used the simple estimate $F_w = \dot\gamma_a(d^2 - \ell^2)$, such that larger quantitative discrepancies are observed, but qualitatively we obtained similar results.

The extra contributions per bundle F_a and F_w have a maximum at $\phi \approx 0.07$ and $\phi \approx 0.09$, respectively, which is close to the results from the point force distribution and the coverage fractions observed in *Xenopus*. A larger coverage would reduce the overall contributions per bundle, both because adding extra bundles will be less effective, with small increments of U per bundle, and there is less space for non-ciliated cells. Decreasing the coverage fraction will on the other end reduce long range transport, as reflected in U; $\dot\gamma_a$ will tend to zero with decreasing ϕ, such that the flow will decay as fast as $1/\rho^3$, as for isolated bundles, and regions in between will not be sheared.

As we concluded for a distribution of point forces, accounting for the finite size of the bundle also leads us to infer that the low coverage fraction observed in *Xenopus* is close to optimal.

Supplementary video. Movie of a cilia bundle and $0.2\,\mu$m diameter tracers, acquired at $2,000$ frames/s, and shown at 30 frames/s. Some larger beads are also present to help visualize the flows.

* fb448@cam.ac.uk
† jerome.jullien@inserm.fr
‡ e.lauga@damtp.cam.ac.uk
§ R.E.Goldstein@damtp.cam.ac.uk

[S1] E. Hörmanseder, A. Simeone, G.E. Allen, C.R. Bradshaw, M. Figlmüller, J. Gurdon, and J. Jullien, H_3K_4 methylation-dependent memory of somatic cell identity inhibits reprogramming and development of nuclear transfer embryos, Cell Stem Cell. **6**, 135 (2017).
[S2] www.enasco.com.
[S3] J. Faber and P.D. Nieuwkoop, Normal Table of *Xenopus laevis* (Daudin) (Garland Publishing Inc., New York, 1994).
[S4] P.R. Bevington and D.K. Robinson, *Data Reduction and Error Analysis for the Physical Sciences* (McGraw-Hill, 1993), 3rd edition.
[S5] M.E. Werner, B.J. Michael, Using *Xenopus* Skin to Study Cilia Development and Function, Methods Enzymol. **525**, (2013).
[S6] T. J. Park B. J. Mitchell, P. B. Abitua, C. Kintner, J. B. Wallingford, Dishevelled controls apical docking and planar polarization of basal bodies in ciliated epithelial cells, Nat. Genet. **40**, 7 (2008)
[S7] J.R. Blake, Note on the image system for a stokeslet in a no-slip boundary, Math. Proc. Camb. Phil. Soc. **70**, 303 (1971).
[S8] F. Boselli, D. Obrist, L. Kleiser. A multilayer method of fundamental solutions for Stokes flow problems, J. Comput. Phys. **231**, 6139 (2012).
[S9] N. Osterman and A. Vilfan, Finding the ciliary beating pattern with optimal efficiency, Proc. Natl. Acad. Sci. USA **108**, 15727 (2011).
